# Transcriptome profiling of the diaphragm in a controlled mechanical ventilation model reveals key genes involved in ventilator-induced diaphragmatic dysfunction

**DOI:** 10.1186/s12864-021-07741-9

**Published:** 2021-06-25

**Authors:** Ruining Liu, Gang Li, Haoli Ma, Xianlong Zhou, Pengcheng Wang, Yan Zhao

**Affiliations:** 1 Emergency Center, Zhongnan Hospital of Wuhan University, 430071 Wuhan, China; 2 Hubei Clinical Research Center for Emergency and Resuscitation, Zhongnan Hospital of Wuhan University, 430071 Wuhan, China; 3 Department of Biological Repositories, Zhongnan Hospital of Wuhan University, 430071 Wuhan, China

**Keywords:** Controlled mechanical ventilation, Ventilator-induced Diaphragmatic dysfunction, RNA-seq, lncRNA, mRNA

## Abstract

**Background:**

Ventilator-induced diaphragmatic dysfunction (VIDD) is associated with weaning difficulties, intensive care unit hospitalization (ICU), infant mortality, and poor long-term clinical outcomes. The expression patterns of long noncoding RNAs (lncRNAs) and mRNAs in the diaphragm in a rat controlled mechanical ventilation (CMV) model, however, remain to be investigated.

**Results:**

The diaphragms of five male Wistar rats in a CMV group and five control Wistar rats were used to explore lncRNA and mRNA expression profiles by RNA-sequencing (RNA-seq). Muscle force measurements and immunofluorescence (IF) staining were used to verify the successful establishment of the CMV model. A total of 906 differentially expressed (DE) lncRNAs and 2,139 DE mRNAs were found in the CMV group. Gene Ontology (GO) and Kyoto Encyclopedia of Genes and Genomes (KEGG) analyses were performed to determine the biological functions or pathways of these DE mRNAs. Our results revealed that these DE mRNAs were related mainly related to complement and coagulation cascades, the PPAR signaling pathway, cholesterol metabolism, cytokine-cytokine receptor interaction, and the AMPK signaling pathway. Some DE lncRNAs and DE mRNAs determined by RNA-seq were validated by quantitative real-time polymerase chain reaction (qRT-PCR), which exhibited trends similar to those observed by RNA-sEq. Co-expression network analysis indicated that three selected muscle atrophy-related mRNAs (*Myog*, *Trim63*, and *Fbxo32*) were coexpressed with relatively newly discovered DE lncRNAs.

**Conclusions:**

This study provides a novel perspective on the molecular mechanism of DE lncRNAs and mRNAs in a CMV model, and indicates that the inflammatory signaling pathway and lipid metabolism may play important roles in the pathophysiological mechanism and progression of VIDD.

**Supplementary Information:**

The online version contains supplementary material available at 10.1186/s12864-021-07741-9.

## Background

Mechanical ventilation (MV) is a life-saving clinical measure for patients who suffer from respiratory failure and are incapable of maintaining alveolar ventilation without assistance [1]. However, the use of controlled mechanical ventilation in both animal and human models causes ventilator-induced diaphragm dysfunction (VIDD) [2], which causes weaning failures in approximately 20 % of patients due to rapid deterioration of diaphragm muscle endurance and strength [3]. The mechanisms of VIDD, however, potentially involve multiple pathological steps, including oxidative stress, muscle atrophy, structural damage, and muscle fiber remodeling, and are not fully clarified [2]. Recent studies have shown that VIDD is characterized mainly by a decline in diaphragmatic contractile force and by muscle atrophy of the diaphragm [4]. It has been demonstrated that complete diaphragm muscle unloading or inactivity following MV induces a profound loss of diaphragm muscle force-generating capacity both *in vitro* [5] and *in vivo* [6, 7]. Prolonged MV is associated with many complications, including poor weaning outcomes, increased intensive care unit (ICU) and hospital mortality, and poor prognosis, thus representing a major medical issue, and it is a heavy burden to patients, their families, and society in general [8]. It has been reported that VIDD occurs in critically ill patients and is characterized by marked diaphragm atrophy of both slow-twitch and fast-twitch fibers [4, 9]. Therefore, it is important to develop a precise understanding of the molecular mechanisms underlying VIDD, especially muscle atrophy, to help ameliorate this important clinical problem.

Long noncoding RNAs (lncRNAs), a novel class of regulatory RNAs commonly defined as transcripts longer than 200 nucleotides, are involved in numerous important biological processes at the transcriptional and posttranscriptional levels [10, 11]. It is known that lncRNAs can act as transcriptional, epigenetic, and translational regulators or perform other functions through interaction with other proteins [12]. Some studies have identified the functions of lncRNAs in skeletal muscle myogenesis and atrophy. The roles of 12 lncRNAs involved in regulating skeletal muscle mass have been explored in many muscle atrophy and hypertrophy models [12]. Hitachi et al. recently reported the role of *Myoparr*, a promoter-associated lncRNA, in myogenesis and skeletal muscle atrophy, which makes this lncRNA a potential target for neurogenic atrophy [13]. *In vitro* and *in vivo* data have demonstrated that mechanical unloading induced muscle atrophy-related lncRNA (*lncMUMA*) can act as a competing endogenous RNA (ceRNA) for miR-762 to regulate *MyoD* abundance, and its overexpression can reverse muscle atrophy in a hindlimb suspension (HLS) model [14]. The lncRNA *MAR1* acts as a ceRNA for miR-487b to regulate the Wnt5a protein during promotion of muscle differentiation and regeneration [15]. Thus, *MAR1* may be associated with muscle atrophy caused by aging or mechanical unloading. *Lnc-mg* (myogenesis-associated lncRNA), which is specifically enriched in skeletal muscle, can promote myogenesis by serving as a ceRNA for miR-351-5p and miR-125b to control insulin-like growth factor 2 protein abundance and regulate β lactamase expression [16, 17]. *lncIRS1* can regulate myoblast proliferation and differentiation *in vitro*, and muscle mass and mean muscle fiber *in vivo*. Furthermore, *lncIRS1* plays a part in regulation of the expression of atrophy-related genes and in muscle atrophy [11].

Myogenin (*Myog*) is a myogenic regulatory factor (MRF). It has been reported that *Myog* expression can exacerbate reductions in muscle force, mass, or cross-sectional area in the denervated soleus muscle [18]. Two muscle-specific E3 ubiquitin ligases, *MuRF1* (muscle RING-finger1), also known as *Trim63*, and MAFBx (muscle atrophy F-box), also known as Atrogin-1 or *Fbxo32*, are the key enzymes of the ubiquitin-proteasome system (UPS) involved in muscle proteolysis and are markers of muscle atrophy in almost all atrophy conditions [19]. *Trim63* and *Fbxo32* mRNA expression has been reported to be elevated in a wide range of atrophy-inducing conditions. *Trim63* is also elevated in human muscle during mechanical ventilation, inflammation, metabolic stress, and COPD, opening up the possibility that both genes could be targets for drug development [20]. *Ppargc1a*, a member of the PPARγ-coactivator 1 (PGC-1) family, has been reported to be a direct target of PPARβ/δ and can regulate muscle fiber type conversion and energy metabolism at the transcriptional level [21]. Myostatin (*Mstn*), also known as growth and differentiation factor 8 (GDF8), is a member of the transforming growth factor-β (TGF-β) superfamily, and is a negative regulator of skeletal muscle growth and development [22]. *Mstn* has been proposed as a biomarker of decreased muscle mass and muscle wasting [23]. It has been found that deletion of *Mstn* induces an increase in skeletal muscle mass [24, 25], while overexpression of *Mstn* can cause dramatic atrophy of skeletal muscle [25, 26]. However, the association between these mRNAs involved in muscle atrophy and lncRNAs have not yet been studied.

Although the functions of some lncRNAs and mRNAs have been partially illustrated, transcriptome profiling of the diaphragm in a CMV model has not been performed to date. Therefore, the aim of our study was to explore the role of lncRNAs in the diaphragm in a CMV model. Thus, in the present study, the expression profiles of lncRNAs and mRNAs in the diaphragms of CMV model rats were explored by high-throughput RNA sequencing. Additionally, we performed GO and KEGG analyses to predict the biological functions and pathways annotated to DE mRNAs. Furthermore, we conducted lncRNA-mRNA co-expression network analysis to explore the potential roles of DE lncRNAs and DE mRNAs following VIDD in a CMV model. The findings may help shed light on the roles of lncRNAs and mRNAs in the pathophysiology underlying VIDD and provide potential new targets for the treatment of VIDD in a CMV model.

## Results

### Establishment of the CMV model

**Fig. 1:**
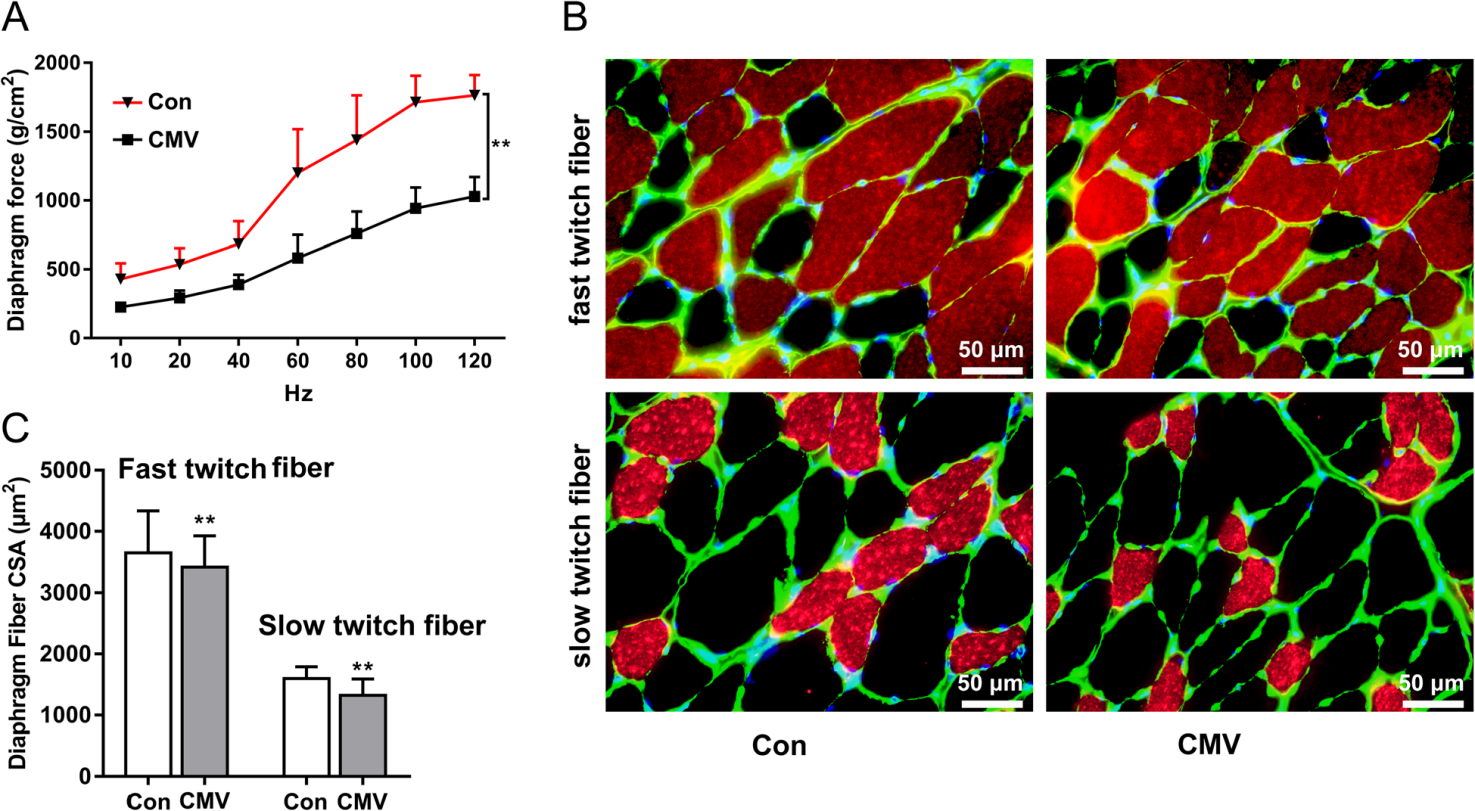
Force-frequency curves, cross-sectional areas and representative immunofluorescence staining images of the diaphragm in all groups. **A** Diaphragm force-frequency curves. **B** Immunofluorescence staining of fast-twitch fibers(top) and slow-twitch fiber (bottom) in the diaphragm, and adjustments that we made were all applied to the entire image; **C** Cross sectional areas (CSAs) of the diaphragm. Scale bar = 50 μm. Groups: Con = control group; CMV = controlled mechanical ventilation group. “**” indicates a *p*-value < 0.01. The error bars indicate the ± SD. All adjustments were applied to the entire image, and adjustments of individual color channels may have caused some changes in the‘merged’images

*In vitro* measurements of muscle strip contractile properties demonstrated a significant reduction in muscle forces in the CMV group compared to control group (Fig. 1A). Immunofluorescence staining showed that the CSA of slow twitch fibers and fast twitch fibers of the diaphragm in the CMV group were significantly decreased after 12 h of CMV (Fig. 1B C). In other words, CMV induced apparent muscle atrophy and the model of CMV was successfully established.

### Mapping of RNA-seq results for the diaphragms of rats after CMV

**Fig. 2:**
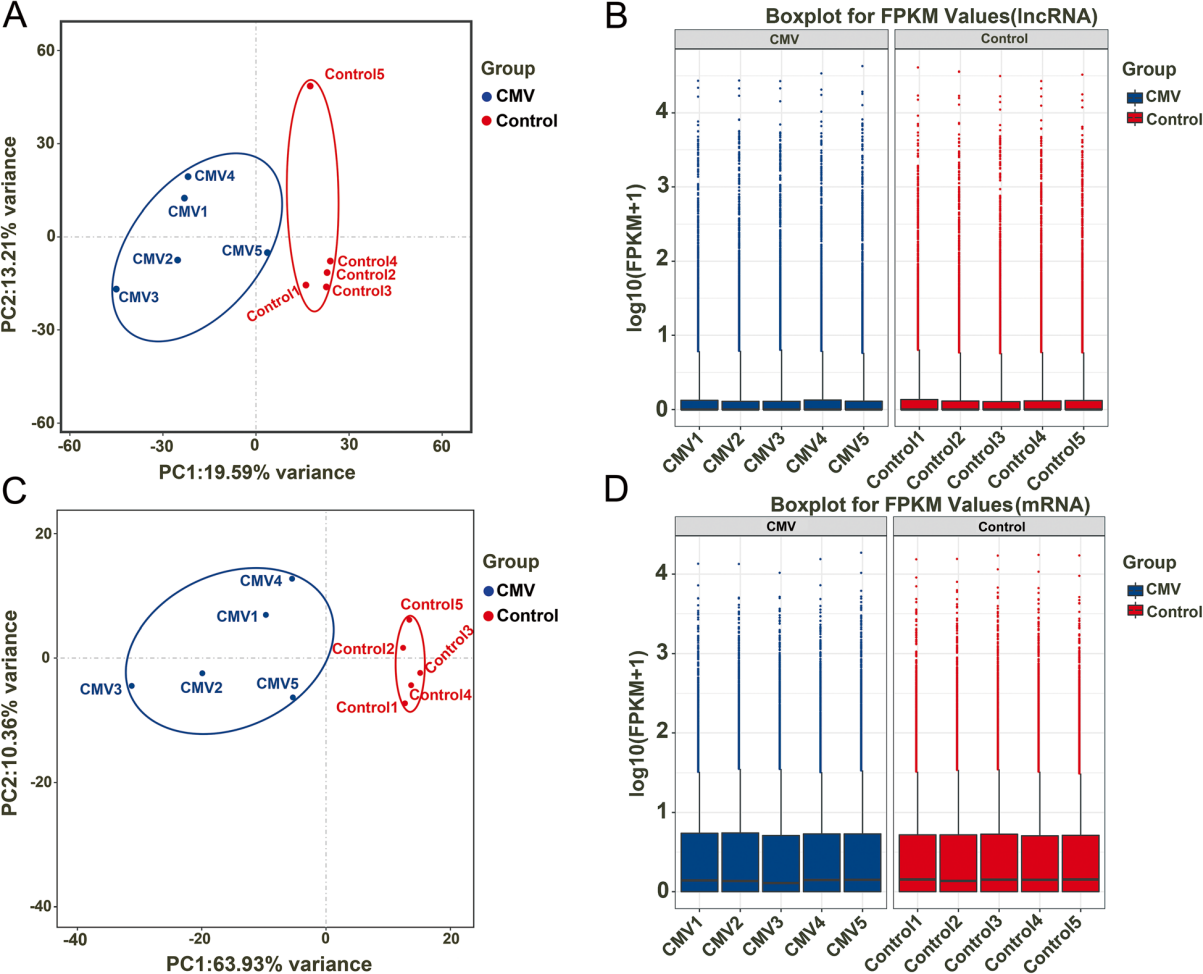
Principal component analysis (PCA) of lncRNAs (**A**) and mRNAs (**C**). Boxplot of the expression levels of 906 DE lncRNAs (**B**) and 2193 DE mRNAs (**D**). The abscissa is the sample name, the ordinate is the log_10_(FPKM + 1), and the boxplot of each area includes five statistics (from top to bottom: maximum, upper quartile, median, lower quartile and minimum)

RNA-seq experiments were performed to discover lncRNAs and mRNAs related to the pathophysiological mechanism of CMV and to compare the transcriptional regulation of samples between the control and CMV groups. All of the raw RNA-seq data have been uploaded to the Sequence Read Archive (SRA), and the accession numbers are indicated in the “Availability of Data and Materials” section. Principal component analysis (PCA) was conducted on the expression level of lncRNAs and mRNAs to investigate the relationships of samples between these two groups (control and CMV) (Fig. 2A and C). We found that the confidence ellipses of samples in the control and CMV groups were separate from each other based on the expression variances of lncRNAs and mRNAs, indicating that the gene expression patterns were similar within the same group and significantly different between the control and CMV groups. Finally, to confirm whether the lncRNA and mRNA expression data could be compared with each other, box-whisker plots were used to show the degrees of dispersion in the expression data distributions (Fig. 2B and D). The distribution of lncRNA and mRNA expression in the control and CMV groups had no obvious differences, indicating that they were suitable for gene expression comparisons. Taken together, these results illustrated that the construction of this CMV model for VIDD was repeatable and that the reliability of these experiments was acceptable for further analysis.

### Differentially expressed lncRNAs in the diaphragm after CMV

**Fig. 3:**
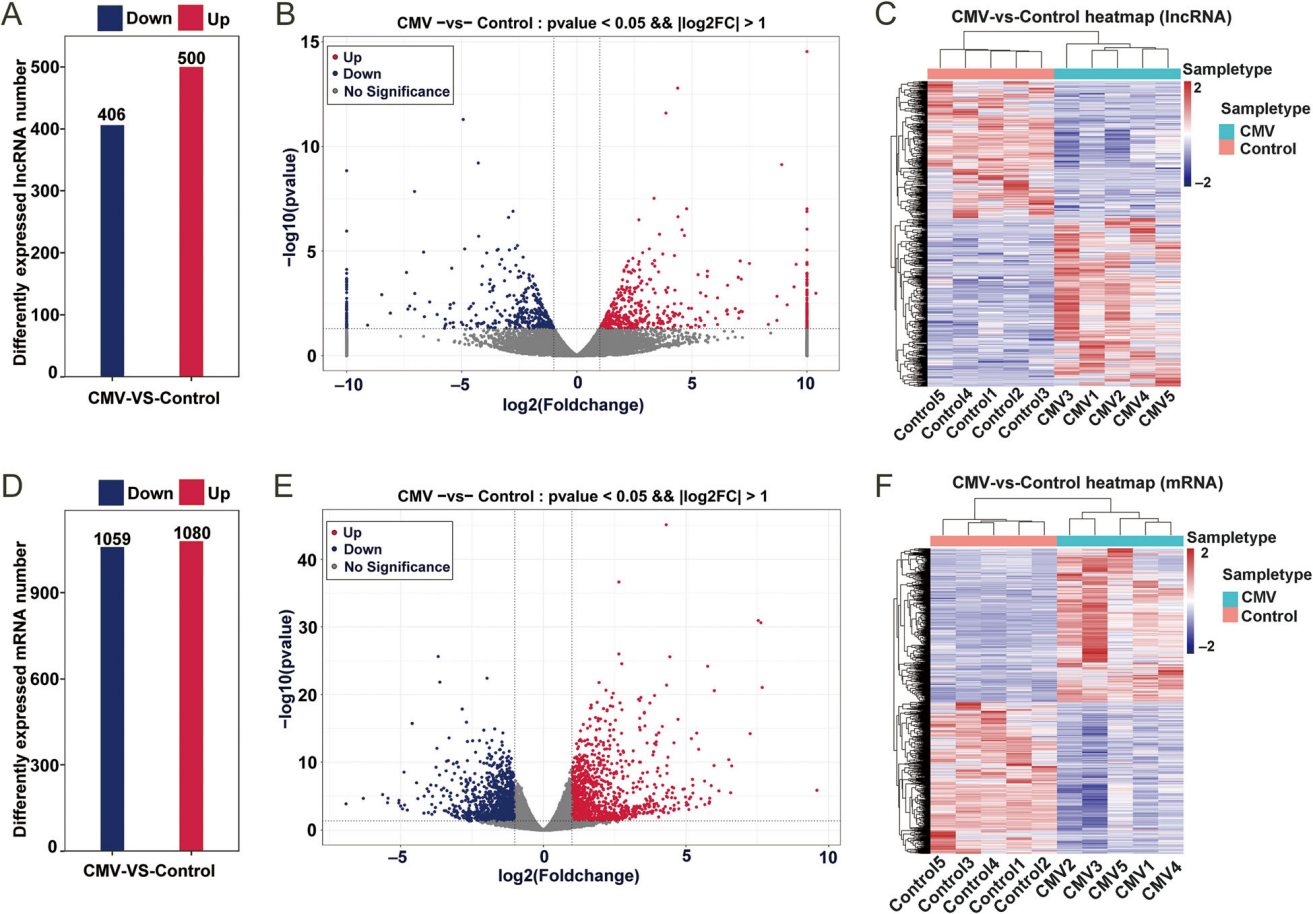
Numbers of up- and downregulated differentially expressed lncRNAs (**A**) and mRNAs (**D**). A volcano plot of the differentially expressed lncRNAs (**B**) and mRNAs (**E**) between CMV and control samples. The top ten differentially expressed lncRNAs and mRNAs are marked. The red points indicate the upregulated lncRNAs or mRNAs, and the blue dots indicate the downregulated lncRNAs or mRNAs. A heatmap of the differentially expressed lncRNAs (**C**) and mRNAs (**F**) between CMV and control samples

We performed multiple testing correction by means of false discovery rate (FDR) calculation with the Benjamini and Hochberg method and found that 129 lncRNAs were obtained when the *p-adj* was < 0.1 and the fold change in the expression level was greater than 2 (Table S2). In the study, the main criteria for screening DE lncRNAs and DE mRNAs were that the raw *p*-value was less than 0.05, and the fold change was greater than 2. In total, 906 DE lncRNAs were identified in the CMV group. A total of 500 were upregulated, while 406 were downregulated (Fig. 3A). The top 10 most significantly differentially upregulated and downregulated lncRNAs based on the *p*-values are listed in Table 1. The most significantly altered lncRNAs were *TCONS_00018037*, *NONRATT021220.2*, *TCONS_00014516*, *TCONS_00018038*, and *NONRATT027286.2* (log2FC values of inf, 4.4, 3.9, 8.9, and 3.4, respectively) in the upregulated group and *NONRATT015099.2*, *NONRATT017141.2*, *TCONS_00003692*, *NONRATT017137.2*, and *TCONS_00014304* (log2FC values of -4.9, -4.3, - inf, -7.0, and − 2.8, respectively) in the downregulated group based on their *p*-values. All DE lncRNAs are displayed using a volcano plot (Fig. 3B) and a heatmap (Fig. 3C). In summary, there were many DE lncRNAs following VIDD in the CMV model, and these lncRNAs may play essential roles in the VIDD response.

**Table 1:** Top10 differentially expressed up-regulated and down-regulated lncRNAs

lncRNA ID	Log_2_FC	*P*-value	Up/down	Chromosome
*TCONS_00018037*	Inf	2.94E-15	Up	Chr20
*NONRATT021220.2*	4.38387522	1.66E-13	Up	Chr4
*TCONS_00014516*	3.87359611	2.52E-12	Up	Chr18
*TCONS_00018038*	8.90230968	7.37E-10	Up	Chr20
*NONRATT027286.2*	3.35575767	3.02E-08	Up	Chr7
*TCONS_00023909*	4.77059451	9.57E-08	Up	Chr5
*NONRATT014418.2*	Inf	9.64E-08	Up	Chr19
*NONRATT011464.2*	Inf	1.29E-07	Up	Chr16
*TCONS_00014514*	4.39529793	2.29E-07	Up	Chr18
*NONRATT017767.2*	2.69916399	3.22E-07	Up	Chr20
*NONRATT015099.2*	-4.934927	2.04E-08	Down	Chr2
*NONRATT017141.2*	-4.2808094	1.97E-06	Down	Chr20
*TCONS_00003692*	- Inf	3.31E-06	Down	Chr1
*NONRATT017137.2*	-7.0496082	2.88E-05	Down	Chr20
*TCONS_00014304*	-2.7718197	0.000159	Down	Chr18
*TCONS_00011884*	-2.965507	0.000266	Down	Chr16
*NONRATT009325.2*	- Inf	0.000944	Down	Chr14
*NONRATT000319.2*	-4.2656254	0.001441	Down	Chr1
*TCONS_00022392*	-2.5775198	0.003859	Down	Chr4
*TCONS_00030445*	-2.6669891	0.004928	Down	Chr8

Gene IDs with “NONRAT” prefix were known long noncoding RNAs which could be found from NONCODE website (www.noncode.org); Gene IDs with “TCONS” prefix were newly identified long noncoding RNAs in this study; *FC* fold change, *Inf* infinity

### Differentially expressed mRNAs in the diaphragm after CMV

There were 2,139 DE mRNAs in the CMV group, with 1,080 being up-regulated and 1,059 being down-regulated (Fig. 3D and Table S3). The top 10 most significantly upregulated and the top 10 most significantly downregulated mRNAs based on the *p*-values are listed in Table 2. The most significantly altered mRNAs were *Pdk4, Cep85l, Mt1, Mt2A*, and *Ifitm1* (log_2_FC values of 4.31, 2.65, 7.53, 7.63, and 2.65, respectively) in the upregulated group and *Nmrk2*, *Acly*, *Pnpla3*, *Papln*, and *Tp53i3* (log_2_FC values of -3.68, -1.97, -3.63, -2.84, and − 2.69, respectively) in the downregulated group. The DE mRNAs are displayed using a volcano plot (Fig. 3E) and a heatmap (Fig. 3F). These DE mRNAs and DE lncRNAs above were further analyzed.

**Table 2:** Top10 differentially expressed up-regulated and down-regulated mRNAs

mRNA ID	Log_2_FC	*P*-value	Up/down	Product
*Pdk4*	4.31	7.60E-46	Up	pyruvate dehydrogenase kinase 4
*Cep85l*	2.65	2.30E-37	Up	centrosomal protein 85-like
*Mt1*	7.53	1.20E-31	Up	metallothionein 1
*Mt2A*	7.63	2.49E-31	Up	metallothionein 2 A
*Ifitm1*	2.65	9.42E-27	Up	interferon induced transmembrane protein1
*Angptl4*	4.43	2.48E-26	Up	angiopoietin-like 4
*Enc1*	2.75	2.65E-25	Up	ectodermal-neural cortex 1
*Hmgcs2*	5.76	6.28E-25	Up	3-hydroxy-3-methylglutaryl-CoA synthase 2
*Acer2*	1.95	1.62E-22	Up	alkaline ceramidase 2
*Hmox1*	4.32	3.88E-22	Up	heme oxygenase 1
*Nmrk2*	-3.68	2.26E-26	Down	nicotinamide riboside kinase 2
*Acly*	-1.97	3.73E-23	Down	ATP citrate lyase
*Pnpla3*	-3.63	1.40E-22	Down	patatin-like phospholipase domain containing 3
*Papln*	-2.84	1.38E-18	Down	papilin, proteoglycan-like sulfated glycoprotein
*Tp53i3*	-2.69	1.23E-16	Down	tumor protein p53 inducible protein 3
*Oxtr*	-4.60	1.84E-16	Down	oxytocin receptor
*Dnah5*	-1.70	1.19E-15	Down	dynein, axonemal, heavy chain 5
*Islr*	-2.09	1.81E-15	Down	immunoglobulin superfamily containing leucine-rich repeat
*Itga6*	-1.42	4.75E-15	Down	integrin subunit alpha 6
*Mss51*	-3.30	2.57E-14	Down	MSS51 mitochondrial translational activator

### GO enrichment and KEGG pathway analyses

**Fig. 4:**
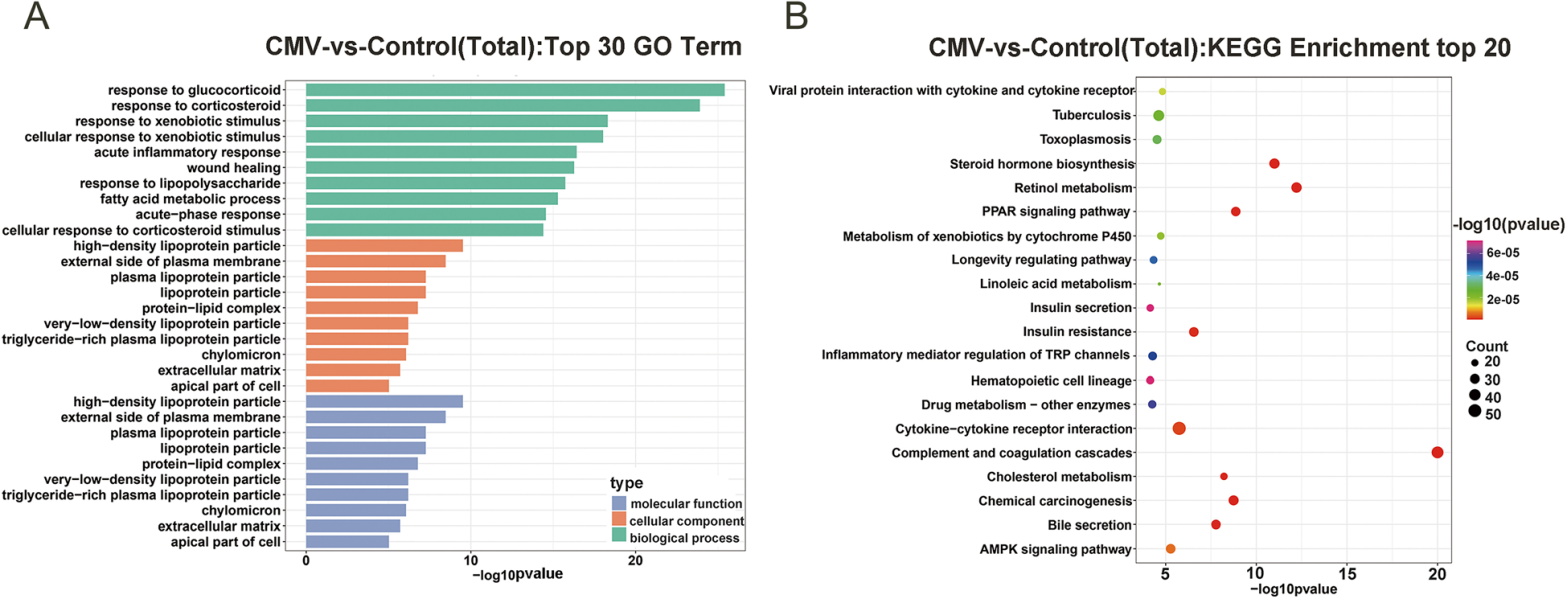
GO and KEGG analyses of DE mRNAs. **a** Top 30 GO terms for the differentially expressed mRNAs in CMV animals and controls. **b** KEGG enrichment analysis of the top 20 differentially expressed mRNAs

To further analyze the DE mRNAs related to VIDD pathophysiology after CMV, GO enrichment analyses were performed. A raw *p*-value < 0.05 indicated the significance of GO term enrichment for DE mRNAs, and the lower the *p*-value was, the more significantly the GO term was enriched in this set. GO enrichment analysis revealed that the 2,139 DE mRNAs in the CMV group were related mainly to the response to glucocorticoid, response to corticosteroid, response to xenobiotic stimulus, cellular response to xenobiotic stimulus, and acute inflammatory response terms in the biological process category; to the high-density lipoprotein particle, external side of plasma membrane, plasma lipoprotein particle, lipoprotein particle, protein-lipid complex terms in the cellular component category; and to the high-density lipoprotein particle, external side of plasma membrane, plasma lipoprotein particle, lipoprotein particle, protein-lipid complex terms in the molecular function category. The top 10 terms in the molecular function (MF) analysis, biological process (BP) analysis, and cellular component (CC) analysis are listed in Fig. 4A.

The KEGG pathway analysis showed that 74 pathways were enriched in these 2,139 DE mRNAs in the CMV group. We also display the top 20 enriched pathways in this work. The top ten most significantly changed pathway terms with the lowest *p-*values were the complement and coagulation cascades, retinol metabolism, steroid hormone biosynthesis, PPAR signaling pathway, chemical carcinogenesis, cholesterol metabolism, bile secretion, insulin resistance, cytokine-cytokine receptor interaction, and AMPK signaling pathway terms (Fig. 4B and Table S4).

### lncRNA and mRNA co-expression network analysis

**Fig. 5:**
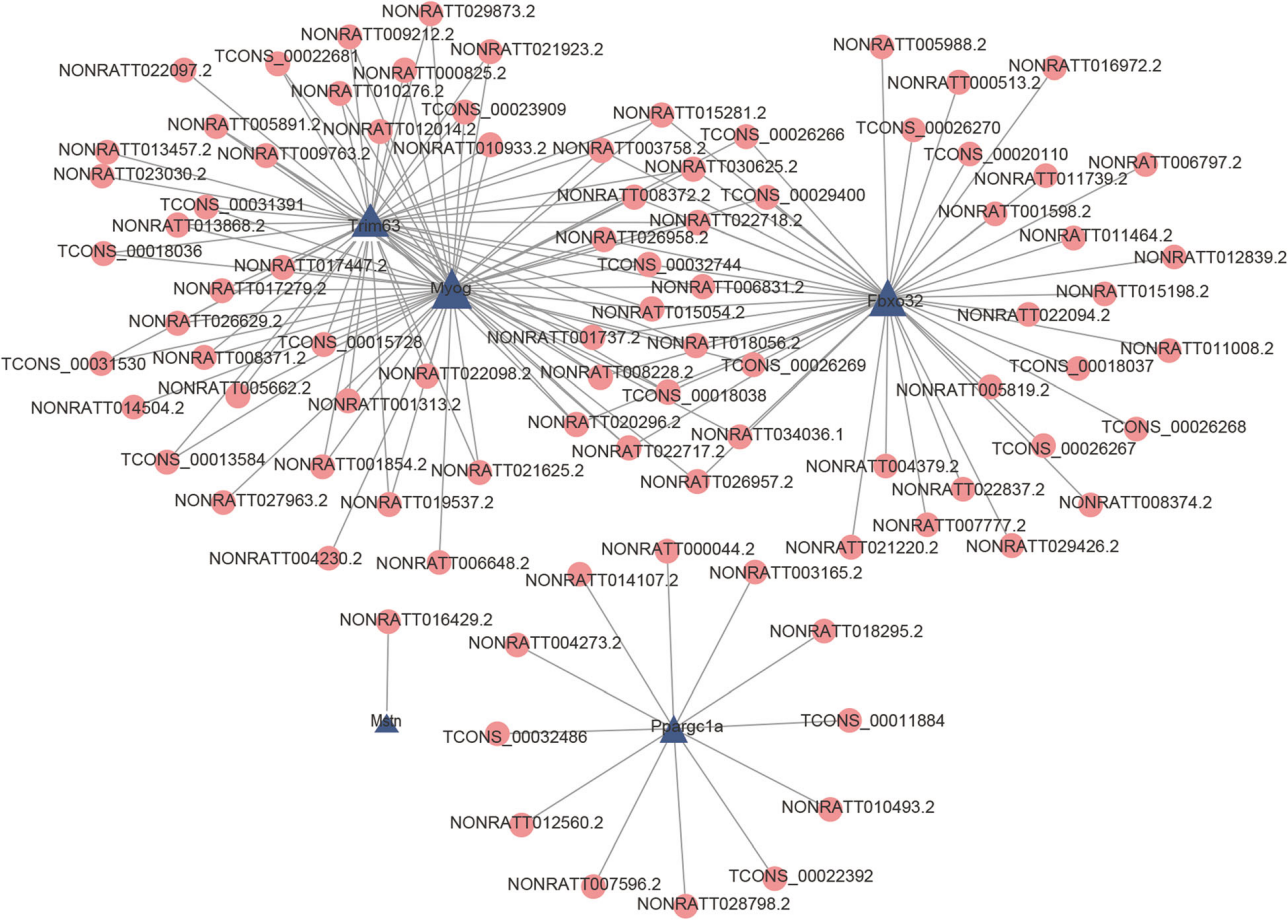
LncRNA-mRNA co-expression network analysis. The blue triangles indicate selected muscle atrophy-related DE mRNAs, and the red circles indicate the DE lncRNAs co-expressed with these mRNAs

To determine which lncRNAs played important roles in the VIDD response, we tried to construct a co-expression network of lncRNAs and mRNAs. First, we found seven mRNAs (*Actn3*, *Fbxo32*, *Gatm*, *Mstn*, *Myog*, *PPARgc1a*, and *Trim63*), annotated as being related to muscle atrophy that were differentially expressed in the CMV group (|log2FC| > 1 and *p* < 0.05) (Table S3). Then, the lncRNA-mRNA pairs with Pearson correlation coefficients > 0.95 and a *p*-value < 0.05 were chosen to construct co-expression network. Finally, five mRNAs (*Myog*, *Trim63*, *Fbxo32*, *Ppargc1a* and *Mstn*) were obtained and used to construct the co-expression network with 90 differentially expressed lncRNAs (Fig. 5). Our results also showed that *Myog* as correlated with 52 lncRNAs, that *Fbxo32* and *Trim63* were correlated with 43 lncRNAs, that *Ppargc1a* was correlated with 12 lncRNAs, and that *Mstn* was correlated with one lncRNA. In addition, as shown by the co-expression network, three mRNAs (*Fbxo32*, *Trim63*, and *Myog*) had more co-expressed lncRNAs than others (Fig. 5). As presented in a Venn diagram (Figure S1), the following 14 lncRNAs overlapped: *NONRATT001737.2*, *NONRATT003758.2*, *NONRATT006831.2*, *NONRATT008228.2*, *TCONS_00032744*, *NONRATT008372.2*, *NONRATT015054.2*, *NONRATT015281.2*, *TCONS_00018038*, *NONRATT018056.2*, *NONRATT020296.2*, *NONRATT022717.2*, *NONRATT022718.2*, and *NONRATT030625.*2, which was presented in a Venn diagram (Figure S1). Therefore, the function of the selected mRNAs were regulated by these lncRNAs, which may play an important role in muscle atrophy but needs further experimentation for verification.

### Quantitative RT-PCR validation

**Fig. 6:**
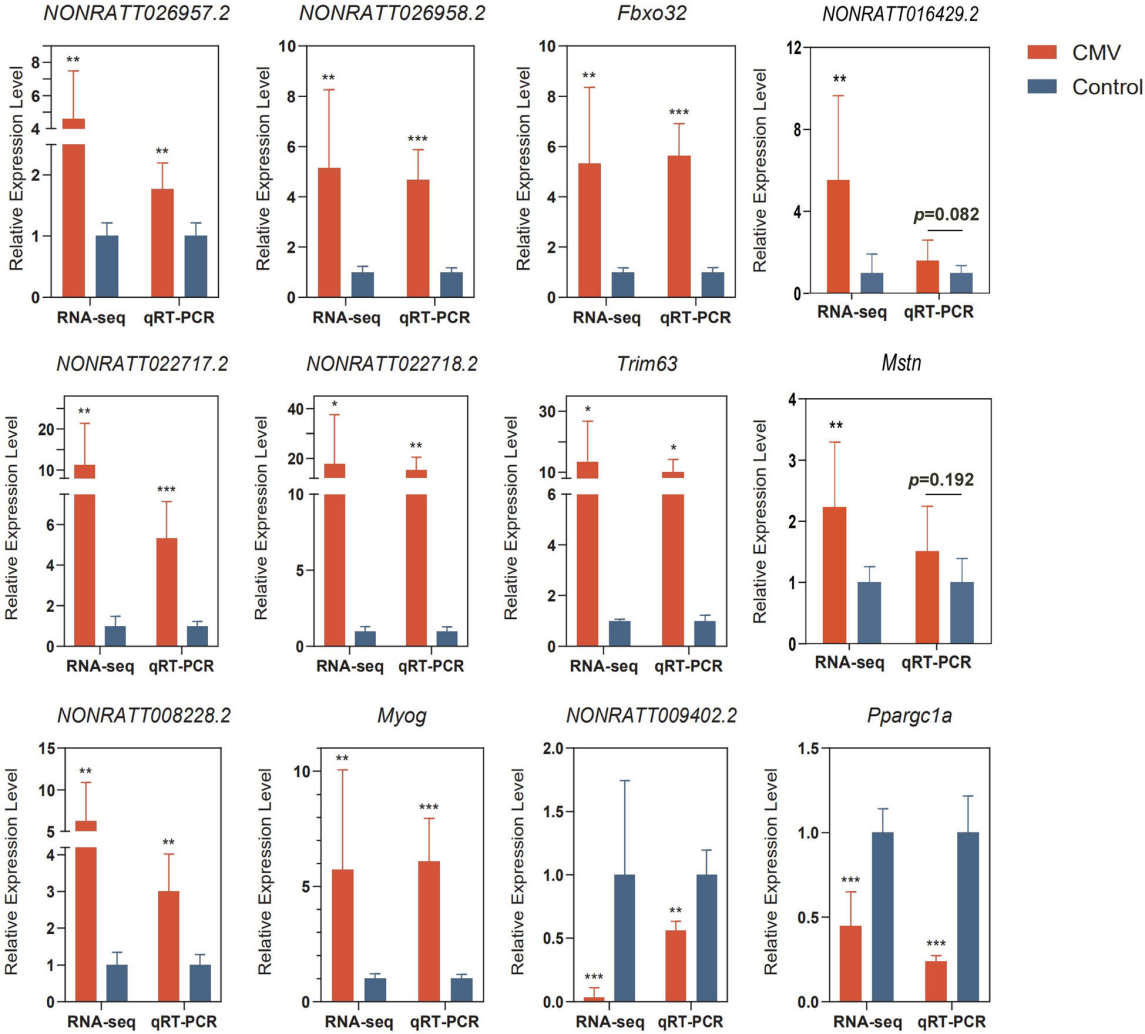
Relative expression level of lncRNAs and mRNAs detected by RNA-sequencing and quantitative real-time PCR (qRT-PCR)

Next, some DE lncRNAs were selected for validation. Comparisons between RNA-seq and qRT-PCR results were performed using independent sample Student’s t tests.We found that *NONRATT008228.2*, *NONRATT026957.2*, *NONRATT026958.2*, *NONRATT022717.2*, and *NONRATT022718.2* were significantly upregulated after CMV,while *NONRATT009402.2* was prominently downregulated after CMV. The five mRNAs (*Myog*, *Trim63*, *Fbxo32*, *Ppargc1a*, and *Mstn*) used to construct co-expression network were also validated by qRT-PCR. The results showed that the expression of *Myog*, *Trim63*, and *Fbxo32* was significantly upregulated, while that of *Ppargc1a* was significantly downregulated. However, there was an upward trend in the expression of *Mstn* and *NONRATT016429.2*, although there were no significant differences (Figures S2 and S3). The expression levels of the top10 upregulated and downregulated DE lncRNAs/mRNAs were partially validated by qRT-PCR. The results were also very consistent with the RNA-seq results, although there was no significant difference about the expression of *NONRATT017137.2*, which was downregulated in the RNA-seq results (Fig. 6, S2, and S3). In summary, the expression patterns of lncRNAs and mRNAs validated by qRT-PCR showed excellent consistency with those detected by RNA-seq.

## Discussion

In this study, we successfully established a CMV model in Wistar rats, and we explored the expression profiles of lncRNAs and mRNAs in the diaphragm using high-throughput RNA-seq in a CMV rat model for the first time. It has been shown that lncRNAs are a diverse type of noncoding RNA containing multiple members with multiple cellular functions. The levels of lncRNAs may regulate myogenic differentiation, myogenesis, and muscle regeneration [13–16]. It has been shown that lncRNAs can regulate multiple aspects of skeletal muscle under health or disease conditions and demonstrate the therapeutic potential for muscular dysfunction [27, 28]. However, there are still many important questions to be addressed.We found that there were 906 DE lncRNAs and 2,139 DE mRNAs that were absent in the control group. Additionally, lncRNA-mRNA network analysis was conducted to further determine the potential roles of DE mRNAs. The findings will likely improve our understanding of the mechanisms and function of DE lncRNAs and DE mRNAs following VIDD in this CMV model.

Emerging evidence suggests that lncRNAs are critical for skeletal muscle physiological and pathological processes [27–32]. To date, many studies have concentrated on the function of lncRNAs in muscle, but our transcriptome profile of the diaphragm in a CMV model is novel. Numerous lncRNAs have been reported and studied in research on myogenesis and muscle regeneration. For example, *lncMyoD*, *lnc-MD1*, *MAR1*, *lnc-mg*, *lnc-YY1*, *Myolinc*, and *Dum* are considered to be important regulators of myogenesis [15, 16, 28–32]. Importantly, impaired myogenesis is a common underlying mechanism of muscle atrophy [33]. Thus, these myogenesis-related lncRNAs may contribute to muscle atrophy. Moreover, some studies have provided clues for further investigation of lncRNAs in muscle atrophy [34]. Some DE lncRNAs and DE mRNAs were validated by qRT-PCR in an independent set of samples. The expression patterns and change trends of these lncRNAs and mRNAs were consistent with those obtained from RNA-seq, although there were no significant differences in the expression of *Mstn*, *NONRATT016429.2* and *NONRATT017137.2*. *Mstn* acts as a negative regulator of skeletal muscle growth, and overexpression of *Mstn* can cause dramatic atrophy of skeletal muscle. In our study, the expression of *Mstn* was slightly upregulated, which may indicate that *Mstn* is not so important in the development of VIDD as other molecules, but more studies are needed to verify its roles in the CMV model. The qRT-PCR validation revealed good consistency and reliability with the observed expression changes detected by RNA-seq. We found that *Myog*, *Trim63*, and *Fbxo32* were significantly upregulated, while *Ppargc1a* was significantly downregulated. The expression patterns and changing trends of these mRNAs are also consistent with what has been reported in other muscle-atrophy models *in vitro* and *in vivo *[18, 19, 35, 36]. We chose some of the genes for validation using qRT-PCR because our objective was to verify the consistency of the qRT-PCR results and RNA-seq results in order to confirm the accuracy and reliability of the RNA-seq results. We also chose five mRNAs (*Myog*, *Trim63*, *Fbxo32*, *Mstn*, and *Ppargc1a*) with known roles in muscle atrophy and explored lncRNAs which with the strongest interactions with these mRNAs based on the Pearson correlation coefficient, and then a co-expression network was constructed. There were 14 co-expressed lncRNAs at the intersection of three mRNAs (*Fbxo32*, *Trim63* and *Myog*), thus, *Fbxo32*, *Trim63*, and *Myog* may be regulated greatly by these 14 co-expressed lncRNAs. It has been reported that these mRNAs have important roles in muscle atrophy, as described below. We speculate that the functions of *Fbxo32*, *Trim63*, and *Myog* are regulated by these lncRNAs, which may have important roles in muscle atrophy following VIDD, but functional validation experiments *in vitro* and *in vivo* are needed to verify the roles. In addition, we cannot rule out the possibility that other DE mRNAs may have more DE lnRNAs with similar gene expression profiles. We wanted to determine which lncRNAs may function with the five known muscle atrophy related mRNAs together in the VIDD response, but we cannot be sure whether these lncRNAs are specific. However, among the differentially expressed lncRNAs, we discovered 90 co-expressed lncRNAs co-expressed with these five mRNAs.

In our RNA-seq and qRT-PCR data, the upregulation of *Myog* may indicate that VIDD contributed to the atrophy of the diaphragm in our CMV model. The expression of *Trim63* and *Fbxo32* in the diaphragm after MV for 12 h was studied in our present study and showed trends similar to those previously reported [36, 37]. The mRNA expression of *Trim63* and *Fbxo32* was elevated within the first 48 h of unloading and inactivity in rodent models. However, there are also several other potential atrophy-related genes, and their particular roles in muscle wasting need to be further clarified in the future [20]. In a very recent study by our team, our results demonstrated that a lung-protective ventilation (LPV) strategy worsened VIDD and that the exacerbation was accompanied by downregulation of PGC-1α in the diaphragm [38]. PGC-1α, an important reactive oxygen species (ROS) inhibitor, plays essential roles in energy metabolism and muscle fiber-type switching [39]. It has been found that the expression of the transcriptional coactivator gene (*Ppargc1a*) is decreased in rats deficient in vitamin D, leading to a loss of skeletal muscle mass and an impaired energy metabolism [40]. Our RNA-seq data also showed that *Ppargc1a* was downregulated, as mentioned above, so we speculated that impaired energy metabolism and decreased muscle fiber content occurred following VIDD. However, further studies are needed to explore the unknown roles of PGC-1α in VIDD. In summary, all of the results above indicate that the RNA-seq results are reliable and valuable and that they can provide us with some clues about the pathophysiology of VIDD following CMV. Of course, there is still much worth studying with regard to the lncRNAs that regulate the functions of essential mRNAs in VIDD in the future.

GO and KEGG pathway analyses were performed to predict the functions of the DE mRNAs. GO enrichment analysis revealed that the DE mRNAs in the CMV group were related mainly to responses to inflammatory signaling pathways and lipoprotein lipase activator activity. In other words, GO and KEGG pathway analysis indicated that the DE mRNAs were associated mainly with inflammation and lipid metabolism. Proinflammatory cytokines are important stimuli that induce muscle atrophy [20, 41–44]. It was initially believed that cytokines may not be contributing factors to the development of VIDD [45]; however, increasing evidence has indicated that increased cytokine mRNA expression can be detected in the human diaphragm muscle after MV and that cytokines may play more important roles in the pathogenesis of VIDD [10]. Additionally, it has been shown that overexpressed IL-6 may be at the center of a signaling network as our RNA-seq results indicated [46, 47]. Notably, generation of inflammatory mediators (IL-6, MIP-2, and TNF-α) can also suppress diaphragmatic contractility [48–51]. We have reported some information about the classic inflammatory signaling pathway in the CMV model together with our RNA-seq results. In our previous research, we found that hemorrhagic shock sensitizes the diaphragm to ventilator-induced atrophy and weakness through activation of IL-6/JAK/STAT signaling-mediated autophagy in rats and that exogenous IL-6 is able to induce activation of JAK/STAT signaling and to increase autophagy in C2C12 cells [52]. Furthermore, it has been shown that the JAK-STAT signaling pathway is the most significantly altered pathway in ventilated human diaphragms for MV times greater than 16 h [51]. However, in our study, the AMPK signaling pathway was significantly altered, which is consistent with some previous research [53, 54]. AMPK activity is a trigger of metabolic events that can activate proteolytic systems and control myofiber size [20]. AMPK has an association with FoxO3 that is involved in protein breakdown and *atrogin-1/MAFbx* expression. However, the metabolic molecular mechanisms involving AMPK signaling in VIDD remain to be elucidated [55].

The pathways detected by our KEGG pathway analysis, such as the PPAR signaling pathway, retinol metabolism pathway, cholesterol metabolism pathway, and insulin resistance pathway, are related to lipid metabolism, as previously reported [56, 57]. It has been shown that diaphragmatic lipid accumulation and responses to master sensors of cellular energy status (AMPK and sirtuins) occur in human MV diaphragms [58, 59]. Lipid accumulation in skeletal muscle may contribute to mitochondrial dysfunction and impaired insulin signaling, thus promoting the development of muscle atrophy. However, the interaction between lipid accumulation and muscle atrophy remains unexplored [60]. Altogether, the evidence indicates that the determining signaling that regulates muscle atrophy may provide potential therapeutic targets for the treatment of VIDD and that lipid metabolism may be a promising research direction in the field of VIDD in the future. There is much work to be done on this topic, and we will explore the potential functions of lncRNAs/mRNAs of interest, the inflammatory signaling pathway and lipid metabolism in the near future.

## Conclusions

In conclusion, our study reveals the transcriptome profile of the diaphragm following CMV for the first time, which may help us to understand the pathophysiological mechanism and progression of VIDD. Importantly, the specific lncRNA expression patterns in the CMV group may also enable the development of disease biomarkers. In addition, the inflammatory signaling pathway and lipid metabolism may be worth studying in the context of VIDD following CMV. However, further studies are needed to develop better MV strategies and pharmacologic agents and to translate the results of basic research into clinical benefits.

## Methods

### Animals

Twenty adult SPF (specific pathogen-free) male Wistar rats weighing 450–550 g were purchased from Charles River Laboratories (Beijing, China). All of the animal studies were performed in the Bio-Safety Level III Laboratory of Wuhan University (Wuhan, Hubei, China). The rats were housed in cages under certain conditions (temperature: 25 °C ± 2 °C; relative humidity: 50 % ± 5 %) with a 12 h:12 h light-dark cycle. Water and food were provided to the rats *ad libitum*. All animal experiments were approved by the Animal Experiment Center and Ethics Committee of Zhongnan Hospital of Wuhan University and followed the National Institutes of Health Guide for the Care and Use of Laboratory Animals.

### Establishment of a Controlled Mechanical Ventilation (CMV) rat model

Twenty Wistar rats were randomly divided into two groups according to a random number table: (1) a control group (*n* = 10), in which the rats received a sham operation with spontaneous breathing, and (2) a controlled mechanical ventilation group (CMV) group (*n* = 10), in which the rats received MV for 12 h with a tidal volume (VT) of 5 ml/kg and a positive end-expiratory pressure (PEEP) of 10 cm H_2_O. This CMV model was established as previously described by our group [1, 38, 61]. Five CMV and five control rats were used for RNA-sequencing, and another ten rats were used for qRT-PCR.

Briefly, the animals were anesthetized with sodium pentobarbital (40 mg/kg body weight) via intraperitoneal injection. The rats were placed and fixed on a heating blanket after anesthesia. Then, the animals were tracheostomized and connected to a volume-driven small animal ventilator (VentElite, Harvard Apparatus, USA). The tidal volume was 5 ml/kg body weight, and the respiratory rate (RR) was set to 55–60 breaths/min. The breathing air was humidified and oxygen-enriched. The ventilator parameters and oxygen flow were adjusted to maintain PaO_2_ at 80–100 mm Hg and PaCO_2_ at 35–45 mm Hg in case of ventilator-induced systemic hypoxia throughout the whole experiment. Arterial blood gas analysis was used to monitor the values of PaO_2_ and PaCO_2_ every 2 h during the experiment. The right jugular vein was cannulated for continuous infusion of normal saline (1 mL/kg/h) and pentobarbital sodium (10 mg/kg/h) using an electric pump. Blood pressure (BP) and heart rate (HR) were monitored through tail cuff plethysmography (BP-2010 Series Blood Pressure Meter, Softron, Japan). The temperature of the rats was maintained at 37 °C during the experiment using a homeothermic blanket system. At the end of the experiment, the rats were euthanized with sodium pentobarbital (100 mg/kg body weight) via intraperitoneal injection for sample collection. The diaphragm samples were stored at -80 °C for further analyses.

### Muscle force measurements

Diaphragm contractile properties were measured as previously described [38, 61]. Each muscle strip was rapidly mounted in a tissue chamber containing Krebs–Henseleit solution (37 °C, pH 7.4), which was bubbled with a gas mixture of 95 % O_2_/5 % CO_2_. The muscle extremities were fixed in spring clips and connected to an electromagnetic force transducer in the experimental apparatus system (ALC-MPA2000, Shanghai, China) for the measurements.

### Immunofluorescence (IF) staining

Immunofluorescence staining was performed on frozen tissues to evaluate the cross-sectional areas (CSAs) of muscle fibers as previously described in our study [38, 61]. Diaphragm tissue embedded in optimal cutting temperature (OCT) were cut into 8-µm slices at -20 °C using a frozen slicer (CM1900, Leica, Germany). Two adjacent sections were stained with either a mouse anti-fast myosin heavy chain antibody (MY-32, Abcam) or an anti-slow myosin heavy chain antibody (NOQ7.5.4D, Abcam). Additionally, rabbit anti-laminin antibody was used to outline the myofibers. Images were obtained using an Olympus IX73-DP80 microscope (SN-3B43822, Olympus Co., Japan), and cellsens standard software were used to capture the image. The wavelength for the excitation filter was 465nm-495nm emitting green light, and the wavelength was 510nm-560nm emitting red light. The CSA were calculated with ImageJ software (Fiji) for at least 200 fibers per animal.

### RNA sequencing for lncRNA and mRNA

Total RNA was extracted from the diaphragms of ten rats (five each for the experimental and control groups) using a mirVana miRNA Isolation Kit (Ambion, Austin, TX, USA) according to the manufacturer’s protocol. RNA integrity was evaluated using an Agilent 2100 Bioanalyzer (Agilent Technologies, USA), and samples with an RNA integrity number (RIN) ≥ 7 were screened for follow-up analysis. A cDNA library covering lncRNAs and mRNAs was constructed using a TruSeq Stranded Total RNA with Ribo-Zero Gold Kit (Illumina, USA) according to the manufacturer’s instructions. The miRNA library was constructed by purifying gel fragments enriched with DNA that was reverse transcribed and amplified from small RNA. Then, these libraries were sequenced on an Illumina sequencing platform (HiSeq 2500), and paired-end reads were generated.

### Data preprocessing and genomic alignment

The raw reads generated from RNA-seq were FASTQ format sequences. To obtain reads with high quality for further analysis, the raw reads were subjected to quality filtering. First, Trimmomatic software was used for adapter removal [62]. Low-quality bases and N-bases or low-quality reads were also filtered out, and high-quality clean reads were obtained. We used HISAT2 to align the clean reads to the reference genome of rats (Rnor_6.0) and the obtained SAM files were used for further analysis.

### lncRNA prediction and expression analysis

The results of alignment to a reference genome were stored in a binary file called a BAM file. StringTie software was used to assemble the reads, and the candidate lncRNA transcripts were screened by comparing the gene annotation information of the reference sequence generated using Cuffcompare software. Finally, transcripts with coding potential were filtered out with CPC [63], CNCI [64], Pfam [65], and PLEK [66] to obtain predicted lncRNA sequences. After aligning the sequencing reads of each specimen with the sequences of mRNA transcripts, known lncRNA sequences and lncRNA sequences were predicted using Bowtie 2, and then the FPKM value and count value (the number of reads for each gene in each specimen) were obtained using eXpress toperform gene quantitative analysis.

### Differential expression analysis

For screening of the differential expression profiles of lncRNAs, we used the estimateSizeFactors function of the DESeq (2012) R package [67] to normalize the counts, and then used the nbinomTest function to calculate *p*-value and fold change values for our difference comparison. The DEmRNAs were analyzed with edgR 3.28.1 software. Differentially expressed transcripts with statistical significance (fold changes ≥ 2 and *p* < 0.05) between the two groups were determined through volcano plot filtering or using a fold change cutoff. The distinguishable expression patterns between samples were determined by hierarchical clustering. Our high-throughput RNA-seq and bioinformatics analyses were performed by Oebiotech, Shanghai, China.

### Gene Ontology annotations and KEGG pathway analyses

The GO and KEGG terms indicating biological roles were enriched with hypergeometric distribution tests to determine the biological functions or pathways that were primarily affected by DE mRNAs. We used clusterProfiler 3.14.3 to analyze the GO and KEGG terms, and the annotation package was org.Rn.eg.db version 3.10.0. The criteria of a *p*-value < 0.05 and a *q*-value < 0.05 were used to identify the significant GO terms associated with the DE mRNAs. The GO terms were derived from the Gene Ontology database (http://geneontology.org/), which comprise three categories: the biological process (BP), cellular component (CC), and molecular function (MF) categories. Next, pathway analysis was performed based on the latest version of the KEGG (https://www.kegg.jp/), which allowed us to determine the molecular interaction and reaction networks of the significantly changed mRNAs. Significant pathways were then chosen to construct a pathway interaction network based on the interaction data in the KEGG database. This pathway relation network was used to identify the pathways that had a regulating effect at the upstream or downstream levels.

### lncRNA-mRNA co-expression network analysis

A lncRNA-mRNA co-expression network was constructed according to the correlations of these DE mRNAs and DE lncRNAs. The genes annotated as being related to muscle atrophy were chosen from the differentially expressed (DE) mRNAs (|log_2_FC|>1 and *p* < 0.05) (Table S3). Then, we explored the lncRNAs that had the strongest interactions with these mRNAs, and the Pearson correlation coefficient of each dysregulated lncRNA-mRNA pair was calculated. Larger values of the Pearson correlation coefficient indicated a stronger interaction. Finally, lncRNA-mRNA pairs with Pearson correlation coefficients > 0.95 and *p*-values < 0.05 were chosen to build co-expression network. This network was constructed for five muscle atrophy-related mRNAs and 90 co-expressed lncRNAs using Cytoscape software (The Cytoscape Consortium, San Diego, CA, USA).

### Quantitative real-time polymerase chain reaction verification in VIDD rats after CMV

Seven DE lncRNAs (*NONRATT009402.2*, *NONRATT026957.2*, *NONRATT026958.2*, *NONRATT008228.2*, *NONRATT022717.2*, *NONRATT022718.2*, and *NONRATT016429.2*) were randomly selected, and the expression levels of these lncRNAs detected by RNA-seq were validated by qRT-PCR using five independent CMV samples and five control samples. These lncRNAs, which target the mRNAs *Myog*, *Ppargc1a*, *Trim63*, *Fbxo32*, and *Mstn*, were also examined with qRT-PCR. Nine lncRNAs and 20 mRNAs among the top10 upregulated and downregulated DE lncRNAs/mRNAs were randomly verified by qRT-PCR. Total RNA was extracted from diaphragm tissue using a mirVana miRNA Isolation Kit (Ambion, Austin, TX, USA) according to the manufacturer’s protocol, and the concentration and purity were assayed using a NanoDrop spectrometer (Thermo Fisher Scientific, Inc.). A total of 1 µg of total RNA was used to synthesize cDNA using a ReverTra Ace qPCR RT Kit (cat. no. FSQ-101; Toyobo Life Science, Osaka, Japan; Code No. FSQ-101). The RNA samples were mixed with RT kit reagents and incubated at 37 °C for 15 min and 99 °C for 5 min. Finally, the samples were diluted in diethylpyrocarbonate-treated water and stored at -20 °C. cDNA was amplified and recorded using SYBR Green Real-Time PCR Master Mix (cat. no. QPK-201; Toyobo Life Science) on a CFX Connect Real-Time PCR System (Bio-Rad; CFX Maestro 1.0 software). All primers were synthesized by Tsingke Biological Technology, Inc., and the sequences are shown in Table S1. The expression of the selected lncRNAs and mRNAs was normalized to that of the housekeeping gene β-actin. The thermocycling program for PCR consisted of 40 cycles of 5 s at 95 °C, 10 s at 55 °C, and 15 s at 72 °C, followed by a final extension at 72 °C for 5 min and a melt curve analysis from 65 to 95 °C. The data were analyzed using the 2^−ΔΔCt^ method [68].

### Statistical analysis

The data are presented as the mean ± standard error of the mean (SEM). Student’s t tests were used for comparisons between two groups. False discovery rates were calculated to correct *p*-values. A |log2FC|>1 and a *p* < 0.05 were used as the thresholds for screening DE lncRNAs and DE mRNAs. All data were analyzed using SPSS version 26.0 software (IBM Corp. Armonk, NY, USA). *p* < 0.05 was considered to indicate statistical significance.

## Supplementary Information

**Additional file 1: Table S1.** Primers designed for qRT-PCR validation of candidate lncRNAs and mRNAs.

**Additional file 2: Table S2.** Detailed information on differentially expressed (DE) lncRNAs (fold change> 2 and *p* < 0.05).

**Additional file 3: Table S3.** Detailed information on differentially expressed (DE) mRNAs (|log2FC|>1and *p*< 0.05).

**Additional file 4: Table S4.** Detailed information on significantly enriched KEGG pathways.

**Additional file 5: Table S5.** The Pearson correlation coefficients of dysregulated lncRNA-mRNA pairs.

**Additional file 6: Figure S1.** Venn diagram showing the intersection of three mRNAs (Fbxo32, Trim63 and Myog) co-expressed with the lncRNAs shown in Fig. 4. The number “14” indicates the following: *NON-RATT001737.2, NONRATT00 3758.2, NONRATT006831.2, NONRATT008228.2, TCONS_00032744, NONRAT T008372.2, NONRATT015054.2, NONRATT015281.2, TCONS_00018038, NON RATT018056.2, NONRATT020296.2, NONRATT022717.2, NONRATT022 718.2*, and *NONRATT030625.2*.

**Additional file 7: Figure S2.** Relative expression levels of the top10 upregulated and down-regulated DE mRNAs detected by RNA-sequencing and quantitative real-time PCR (qRT-PCR).

**Additional file 8: Figure S3.** The relative expression levels of nine lncRNAs among the top 10 upregulated and downregulated DE lncRNAs detected by RNA-sequencing and quantitative real-time PCR (qRT-PCR).

## Data Availability

All of the raw data have been uploaded to the Sequence Read Archive (SRA), and the accession numbers are SRR13083405-13083414. The data can be accessed at https://www.ncbi.nlm.nih.gov//bioproject/PRJNA678858. The lncRNA database we refer to can be found at http://www.noncode.org/, the Genome Database can be found at ftp://ftp.ncbi.nlm.nih.gov/genomes/all/GCF/000/001/895/GCF_000001895.5_Rnor_6.0/GCF_000001895.5_Rnor_6.0_genomic.fna.gz, and the mRNA Database can be found at ftp://ftp.ncbi.nlm.nih.gov/genomes/all/GCF/000/001/895/GCF_000001895.5_Rnor_6.0/GCF_000001895.5_Rnor_6.0_rna.fna.gz.

## Abbreviations

CMV: Controlled mechanical ventilation
VIDD: Ventilator-induced diaphragmatic dysfunction
lncRNA: Long non-coding RNA
RNA-seq: RNA-sequencing
GO: Gene Ontology
KEGG: Kyoto Encyclopedia of Genes and Genomes
RT-qPCR: Reverse transcription-quantitative PCR
HLS: Hindlimb suspension
ceRNA: Competing endogenous RNA
*Lnc-mg*: Myogenesis-associated lncRNA
MF: Molecular function
BP: Biological process
CC: Cellular component
PGC-1: PPARγ-coactivator 1
*Myog*: Myogenin
MRFs: Myogenic regulatory factors
*MuRF1*: Muscle RING finger-1
*MAFBx*: Muscle atrophy F-box
UPS: Ubiquitin-proteasome system
LPV: Lung-protective ventilation
ROS: Reactive oxygen species
VT: Tidal volume
PEEP: Positive end-expiratory pressure
RR: Respiratory rate
BP: Blood pressure
HR: Heart rate
