# Supplementary material for: Transcriptome profiling of the diaphragm in a controlled mechanical ventilation model reveals key genes involved in ventilator-induced diaphragmatic dysfunction
Source: BMC Genomics. 2021 Jun 25;22:472. doi: 10.1186/s12864-021-07741-9 (PMC8227366; doi:10.1186/s12864-021-07741-9)
Supplement: Supplementary file 1 — Additional file 1: Table S1. Primers designed for qRT-PCR validation of candidate lncRNAs and mRNAs. [file 12864_2021_7741_MOESM1_ESM.docx]

**Table S1**. Primers designed for qRT-PCR validation of candidate lncRNAs and mRNAs

| **lncRNA/mRNA** | **Primer type** | **Primer Sequence (5’-3’)** |
| --- | --- | --- |
| NONRATT008228.2 | Forward | GGCAAGCATTTGCAGTGGAT |
| NONRATT008228.2 | Reverse | GCATCAGCACAGGAGACCTT |
| NONRATT026957.2 | Forward | ATGGCCACATCCAAACTCCAA |
| NONRATT026957.2 | Reverse | TCCGAAAGCTCCCAAGTCTG |
| NONRATT026958.2 | Forward | CAGGCTCCTTATTTCTGCCGT |
| NONRATT026958.2 | Reverse | TACACTGCTAACCAACTCCTGT |
| NONRATT022717.2 | Forward | TGTCCACTCTGTAGTCCCAGA |
| NONRATT022717.2 | Reverse | CTACAGCTTACTCCGTGGCAG |
| NONRATT022718.2 | Forward | GGTGACTGGAATCTGAAGGGT |
| NONRATT022718.2 | Reverse | GACCGGAGATGCCTGACATT |
| NONRATT009402.2 | Forward | CATTCAAAGCAGCAGCGAGG |
| NONRATT009402.2 | Reverse | AGTTCTCGGTACAGCCATCA |
| NONRATT016429.2 | Forward | TGTGCATGACTAAACAGTGCC |
| NONRATT016429.2 | Reverse | GGAATTCTCCCAAAGTGTGGAC |
| TCONS_00018037 | Forward | TTGATCTCCAACTGCCGCCT |
| TCONS_00018037 | Reverse | GAGAACCGGTCAGGGTTATGC |
| NONRATT021220.2 | Forward | CTGAGGTGCTTCTGGTTGTGA |
| NONRATT021220.2 | Reverse | CCAAATGCCGAGAGTTTGTGT |
| TCONS_00014516 | Forward | ACTGTCTTCTCCAGCCAAGC |
| TCONS_00014516 | Reverse | AAGAGACAGTTGCTCCCGTG |
| TCONS_00018038 | Forward | TTCCTGCTGCGCCTGAAG |
| TCONS_00018038 | Reverse | GTGTGGAGAACCGGTCAGG |
| NONRATT027286.2 | Forward | GAGAGTGAAGATGCGGAAGGT |
| NONRATT027286.2 | Reverse | CACAACCTTCAAATGCACGGA |
| NONRATT015099.2 | Forward | CACCACTGTGAGGAGGTAGC |
| NONRATT015099.2 | Reverse | TGTTCATCCCATAGGCCTGC |
| NONRATT017141.2 | Forward | CTGCCTGTGTTCCCTACCAA |
| NONRATT017141.2 | Reverse | CTCAAGCTCAGGGTAGCAGG |
| NONRATT017137.2 | Forward | TTCCCTTCCACAGCCAAGTC |
| NONRATT017137.2 | Reverse | AGCCATCCACACTTGTGTCA |
| TCONS_00014304 | Forward | CAAATGGAGGATGGGCTGCT |
| TCONS_00014304 | Reverse | TGGCGGACATTACCTACCCA |
| β-actin | Forward | AGATGTGGATCAGCAAGCAGGAG |
| β-actin | Reverse | CGCAAGTTAGGTTTTGTCAAGAAACC |
| Myog | Forward | TCCAGTACATTGAGCGCCTA |
| Myog | Reverse | CACGATGGACGTAAGGGAGTG |
| Trim63 | Forward | TGGGGAAGACAGAACAAGGC |
| Trim63 | Reverse | ATTGGTGTCCCTCTGTGGAC |
| Fbxo32 | Forward | AGCTTGTGCGATGTTACCCA |
| Fbxo32 | Reverse | GGTGAAAGTGAGACGGAGCA |
| Ppargc1a | Forward | GAGGGACGAATACCGCAGAG |
| Ppargc1a | Reverse | CTCTCAGTTCTGTCCGCGTT |
| Mstn | Forward | CACGCTACCACGGAAACAATC |
| Mstn | Reverse | TCCACAGCTGGGCCTTTAC |
| Pdk4 | Forward | GTATCGACCCCAACTGCGAT |
| Pdk4 | Reverse | GGCGGTTTTCTTGATGCTCG |
| Cep85l | Forward | GGGATAATGAACTACGGGCG |
| Cep85l | Reverse | GGCCTGAACTGCTCTCGTAT |
| Mt1 | Forward | GTGCCTGAAGTGACGAACAG |
| Mt1 | Reverse | CACATGCTCGGTAGAAAACGG |
| Mt2A | Forward | GGCTCCTGCAAATGCAAACA |
| Mt2A | Reverse | GCACTTGTCCGAAGCCTCTT |
| Ifitm1 | Forward | GGACCAAGCCTGTATCCTCAAA |
| Ifitm1 | Reverse | TTGATTGTGGTGGTTGTCGC |
| Angptl4 | Forward | CTGGGACCAAGACCACGAC |
| Angptl4 | Reverse | GCCGTTGCCGTGGAATAGAG |
| Enc1 | Forward | CTCTGATCCCTACCGCGTTC |
| Enc1 | Reverse | TCGTGTGTGTCAGAGTGAGC |
| Hmgcs2 | Forward | AGGAGGCCAATCCATACAACC |
| Hmgcs2 | Reverse | GATCCTATGGGGTCGCTGTG |
| Acer2 | Forward | GTGCTTTGGCCATGTGGTTT |
| Acer2 | Reverse | ACGCCAGGCATGTTGTAACT |
| Hmox1 | Forward | AAGAGGCTAAGACCGCCTTC |
| Hmox1 | Reverse | CCTCTGGCGAAGAAACTCTGT |
| Nmrk2 | Forward | CACCTCAGGACCAGTCAACA |
| Nmrk2 | Reverse | GCACGTCCCACTGTTTGAAG |
| Acly | Forward | TACGGACAGAGAGCCACACT |
| Acly | Reverse | GTCTGTGTCGGGAGTAACCC |
| Pnpla3 | Forward | TCACCTTCGTGTGCAGTCTC |
| Pnpla3 | Reverse | TTCTCCCCATCGGACACTCT |
| Papln | Forward | CTTCGTGAGGCAGACAGAGATG |
| Papln | Reverse | TCCGTCTCTCCTCTGGGAGTA |
| Tp53i3 | Forward | GGGCAGGATTGTGGTGTTG |
| Tp53i3 | Reverse | GCTGCATCACATGAAGGAAGG |
| Oxtr | Forward | GGTCACTCAGCCATCAGAGG |
| Oxtr | Reverse | ATTAGCACCACTCTGCGTCC |
| Dnah5 | Forward | GAACTACATCGCTGCTGTGGA |
| Dnah5 | Reverse | AGCATCCAAGGAAGTGTATGGG |
| Islr | Forward | GAAAGAGCTGTGTGCAAAGAAGAA |
| Islr | Reverse | GTCTAGGTCACGATAGGCACA |
| Itga6 | Forward | TTCGGCTACTCAATCGCTGG |
| Itga6 | Reverse | ATGGATTTCTGGCGGAGGTC |
| Mss51 | Forward | ACCGTGTCATGGAATGGCTT |
| Mss51 | Reverse | GACATCTGGGTCTGGTCGTG |
