# Supplementary figures and images for: Transcriptome profiling of the diaphragm in a controlled mechanical ventilation model reveals key genes involved in ventilator-induced diaphragmatic dysfunction
Source: BMC Genomics. 2021 Jun 25;22:472. doi: 10.1186/s12864-021-07741-9 (PMC8227366; doi:10.1186/s12864-021-07741-9)

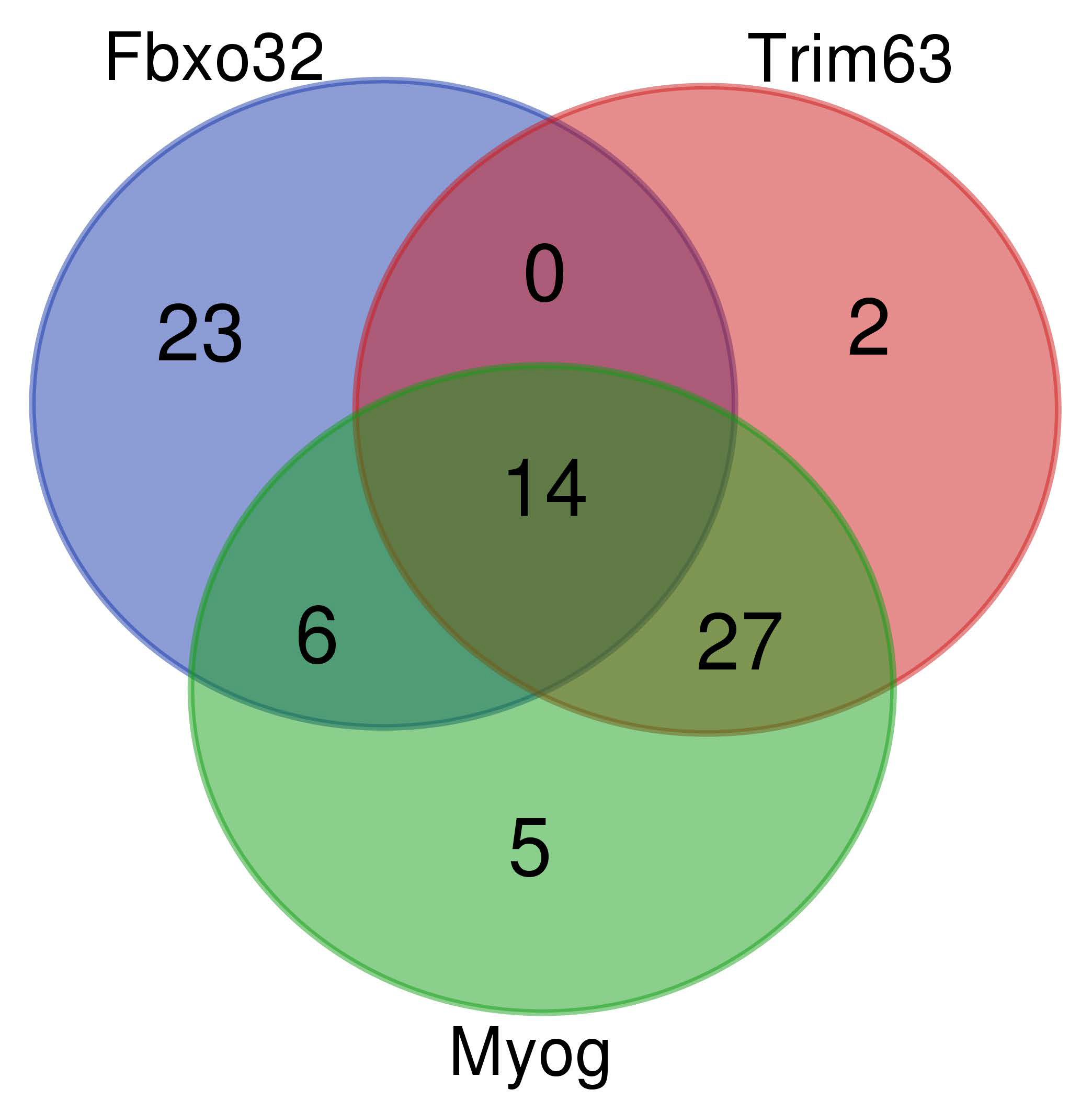

Supplement: Supplementary file 6 — Additional file 6: Figure S1. Venn diagram showing the intersection of three mRNAs (Fbxo32, Trim63 and Myog) co-expressed with the lncRNAs shown in Fig. 4. The number “14” indicates the following: NON-RATT001737.2, NONRATT00 3758.2, NONRATT006831.2, NONRATT008228.2, TCONS_00032744, NONRAT T008372.2, NONRATT015054.2, NONRATT015281.2, TCONS_00018038, NON RATT018056.2, NONRATT020296.2, NONRATT022717.2, NONRATT022 718.2, and NONRATT030625.2. [file 12864_2021_7741_MOESM6_ESM.tif]

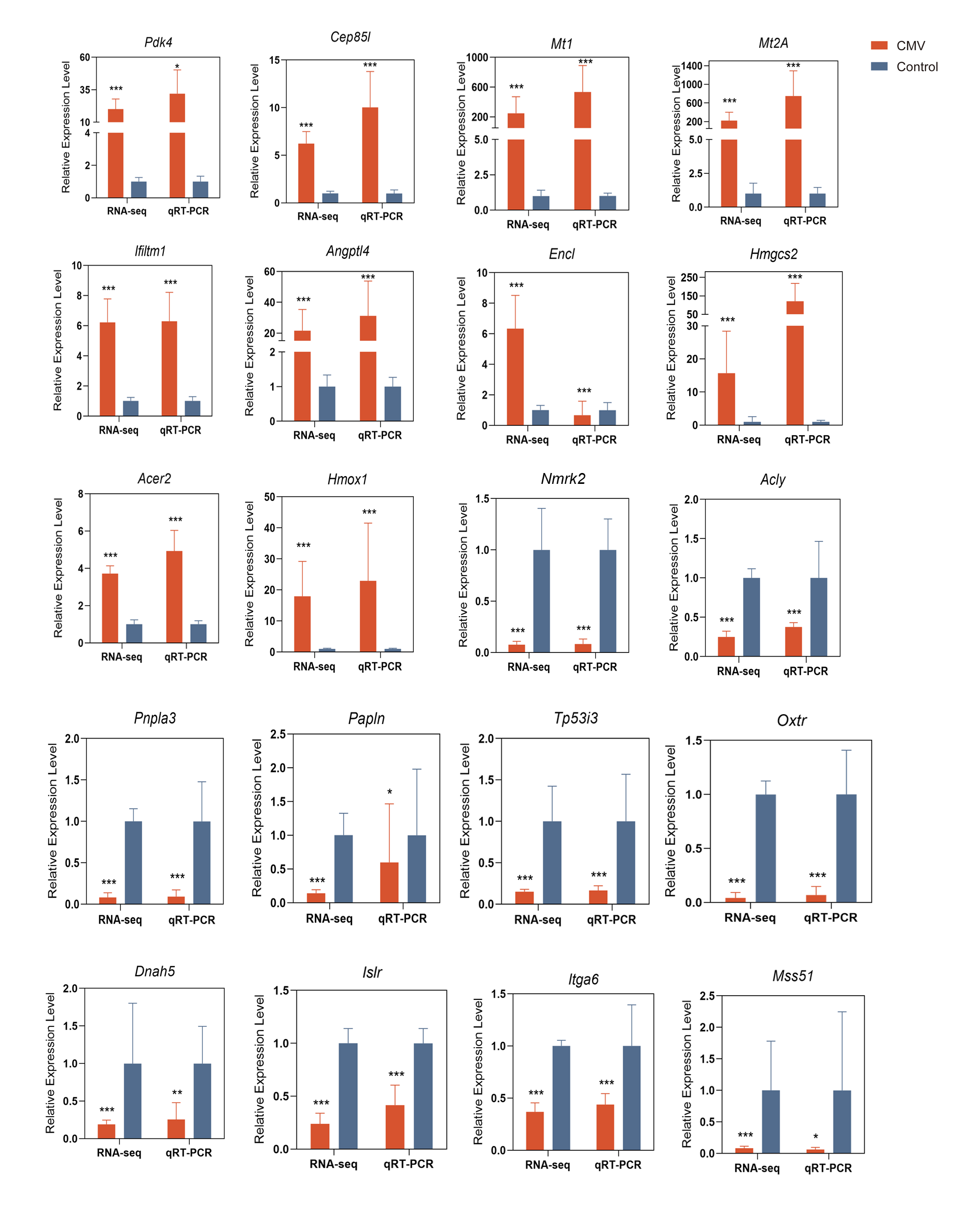

Supplement: Supplementary file 7 — Additional file 7: Figure S2. Relative expression levels of the top10 upregulated and down-regulated DE mRNAs detected by RNA-sequencing and quantitative real-time PCR (qRT-PCR). [file 12864_2021_7741_MOESM7_ESM.tif]

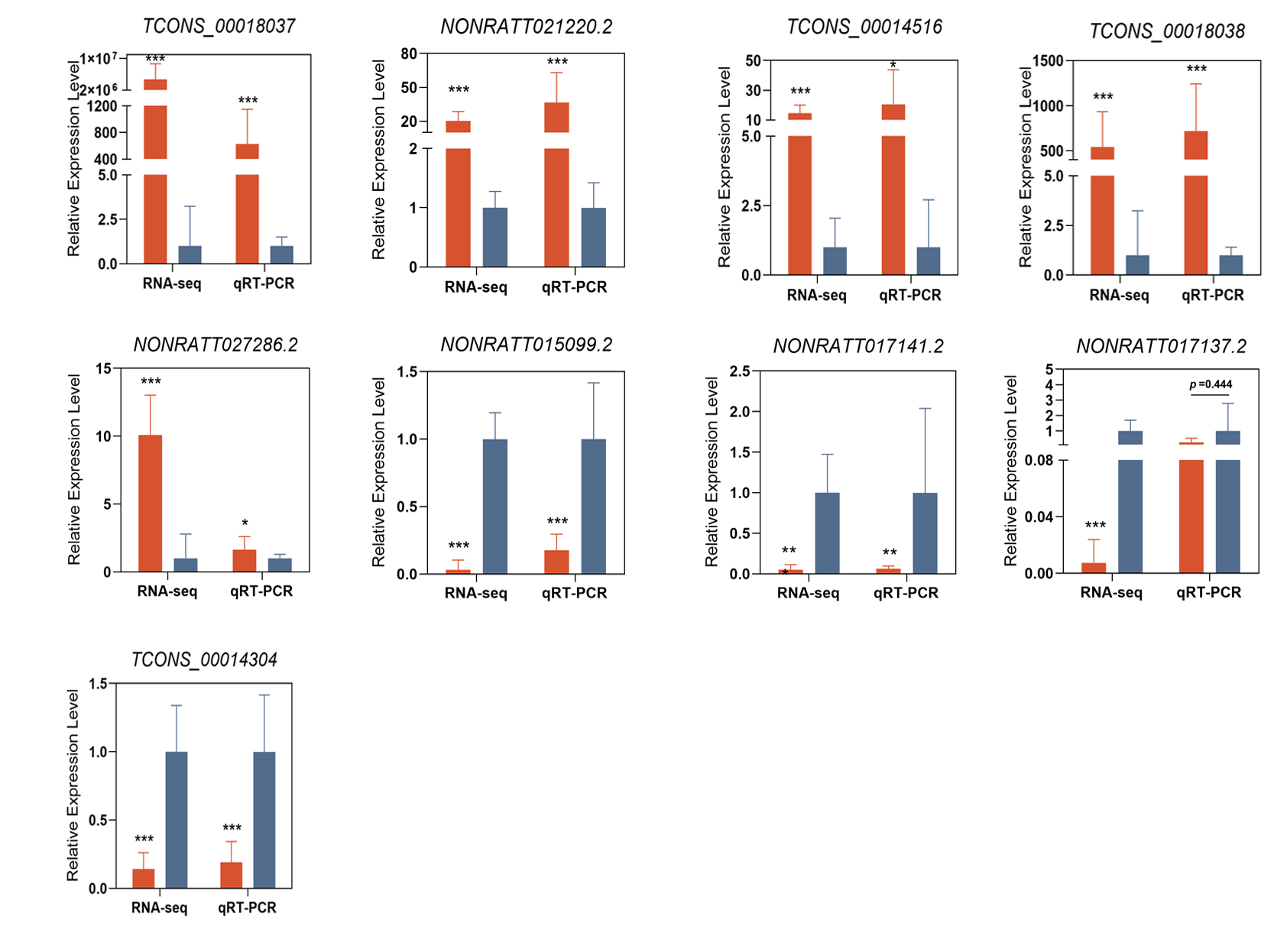

Supplement: Supplementary file 8 — Additional file 8: Figure S3. The relative expression levels of nine lncRNAs among the top 10 upregulated and downregulated DE lncRNAs detected by RNA-sequencing and quantitative real-time PCR (qRT-PCR). [file 12864_2021_7741_MOESM8_ESM.tif]
